# Demethylating drugs alter protoplast development, regeneration, and the genome stability of protoplast-derived regenerants of cabbage

**DOI:** 10.1186/s12870-025-06473-2

**Published:** 2025-04-11

**Authors:** Agnieszka Kiełkowska, Agnieszka Brąszewska

**Affiliations:** 1 Department of Plant Biology and Biotechnology, Faculty of Biotechnology and Horticulture, University of Agriculture in Krakow, Al. 29-Listopada 54, Krakow, 31-425 Poland; 2 Institute of Biology, Biotechnology and Environmental Protection, Faculty of Natural Sciences, University of Silesia in Katowice, 28 Jagiellonska Street, Katowice, 40-032 Poland

**Keywords:** Cabbage, DNA hypomethylation, Methyltransferase inhibitors, Mitotic divisions, Protoplasts, Ploidy, Regeneration

## Abstract

**Background:**

Methylation is a major DNA modification contributing to the epigenetic regulation of nuclear gene expression and genome stability. DNA methyltransferases (DNMT) inhibitors are widely used in epigenetic and cancer research, but their biological effects and the mechanisms of their action are not well recognized in plants. This research focuses on comparing the effects of two DNMT inhibitors, namely 5-azacytidine (AZA) and zebularine (ZEB), on cellular processes, including organogenesis in vitro. Protoplasts are a unique single-cell system to analyze biological processes in plants; therefore in our study, both inhibitors were applied to protoplast culture medium or the medium used for the regeneration of protoplast-derived calluses.

**Results:**

AZA induced a dose-dependent reduction in protoplast viability, delayed cell wall reconstruction, and reduced mitotic activity, while ZEB in low concentration (2.5 µM) promoted mitoses and stimulated protoplast-derived callus development. The higher effectiveness of shoot regeneration was observed when drugs were applied directly to protoplasts compared to protoplast-derived callus treatments. Our findings reveal that both drugs affected the genome stability of the obtained regenerants by inducing polyploidization. Both drugs induced hypomethylation and modulated the distribution patterns of methylated DNA in the protoplast-derived callus.

**Conclusion:**

AZA was more toxic to plant protoplasts compared to ZEB. Both inhibitors affect the ploidy status of protoplast-derived regenerants. A comparison of the data on global methylation levels with the regeneration efficiency suggests that organogenesis in cabbage is partially controlled by variations in DNA methylation levels.

## Background

Plant protoplasts are complete cells that have undergone the removal of the cell wall through enzymatic treatment. As a result, the plasma membrane serves as the sole barrier between the cytoplasm and the external environment. Protoplasts are a versatile single-cell system for genomics [1, 2], transcriptomics [3], proteomics [4], and metabolomics studies [5], as well as new plant breeding technologies, such as CRISPR-Cas-mediated transformation [2, 6]. The research on protoplasts started in the 60s of the past century, but an efficient protoplast-to-plant system is available only for some species, including *Brassica napus*, *Nicotiana* sp., *Citrus* sp., *Daucus carota*, or *Solanum* sp [6–10]. In many economically important species, the practical potential of the protoplasts (i.e., somatic hybridization, cybrids, transformation) is not utilized due to the low or absence of plant regeneration.

*Brassica oleracea* is a diploid (CC genome, 2n = 2x = 18) crop species, exhibiting enormous phenotypic variations, i.e., cabbage, cauliflower, Brussels sprouts, kale, savoy, kohlrabi, or broccoli. Cabbage is considered the most important cool-season crop worldwide. It is the fourth most produced vegetable in the world, after tomatoes, onions, and cucumbers; the major producers are Asia and Europe [11]. Cabbages have dietetic and nutritional benefits and anti-cancer properties [12]. In cabbage, plant regeneration from protoplast-derived cells and somatic fusion has been reported; however, the success was achieved mainly for the superior genotypes [13–16]. Although much effort has been devoted to the development of efficient protocols for protoplast culture in cabbage, plant regeneration remains the primary obstacle in the practical implementation of protoplast technology. Therefore, any enhancements increasing the efficacy of regeneration in cabbage are of great value.

Studies on microspore- and somatic embryogenesis have shown that induced epigenetic modifications regulate the activity of genes involved in these processes, which affects their final efficiency [17–19]. Protoplast studies in this regard are sparse, but the available results suggest that cell competency may be controlled by changes in DNA methylation [20, 21]. DNA methylation refers to the addition of a methyl group (-CH_3_) at the C-5 or N-4 positions of cytosine, and at the N-6 position of adenine [22]. This process, next to histone modifications and non-coding RNA, contributes to the epigenetic regulation of nuclear gene expression and genome stability [23]. In both plants and mammals, DNA methylation is catalyzed by conserved DNA methyltransferases (DNMT) using *S*-adenosyl-l-methionine as the methyl donor, whereas active DNA demethylation involves a base excision repair pathway [24]. Precise patterns of genomic DNA methylation are conserved in plants and are crucial to many biological processes. DNA methylation and/or demethylation levels in different tissues or cell types are tightly controlled during growth and development and throughout a plant’s life cycle [25], but they also regulate many important genes for stress responses in plants ie. *HvMET*, *HvCMT*, and *HvDME* genes involved in the DNA methylation or demethylation machineries under water deficiency in barley [26] or *BramMDH1*, *BraKAT2*, *BraSHM4*, and *Bra4CL2* genes involved epigenetic regulation under cold stress in *Brassica rapa* [27]. Recently, chemical demethylation has gained interest due to its epigenetic reprogramming ability during cell differentiation [19, 28]. The chemical demethylation is based on the use of chemical agents, i.e., 5-azacytidine (AZA), the AZA derivative 5-aza-2’-deoxycytidine (DAC), or zebularine (ZEB), acting as nonmethylable cytosine analogs, incorporating into the DNA double helix in the place of cytosine with each cycle of DNA replication [29]. These nucleoside analogs were originally developed as cancer chemotherapeutics and are powerful inducers of genes silenced by DNA methylation [30]. AZA covalently binds to DNMT, forming nucleoprotein adducts, which depletes the number of active DNA methyltransferase enzymes in the cell [31]. In 2002, AZA was approved by the Food and Drug Administration (USA) under the name Vidaza^®^, as a legal drug for cancer treatments [32, 33]. ZEB is a cytidine deaminase inhibitor, forming tight covalent complexes with DNMT enzymes, resulting in reduced genomic DNA methylation [34]. ZEB was developed to counter the shortcomings of AZA and DAC, in particular, cytotoxicity and instability in aqueous solutions [30, 35, 36] and its undergoing clinical stage investigations [37]. The effect of AZA and DAC on somatic embryogenesis [38], microspore embryogenesis [17, 19], or abiotic stress tolerance [39] has been evaluated in plants; however, the effect of ZEB on plant developmental processes is unexplored. It has been shown that ZEB induces dose-dependent and transient growth inhibition in *Arabidopsis* and *Medicago sativa* seedlings [40], and increases anthocyanin accumulation in grapevine [41].

The aim of our study was to verify the hypothesis stating that the modification of DNA methylation level, caused by DNMT inhibitors, modulates the mitotic activity of protoplasts and the efficiency of plant regeneration. We analyzed the effect of the DNMT inhibitors (ZEB and AZA) on various cellular processes (viability, cell wall regeneration, mitotic activity, efficiency of callus production, and organogenesis from callus). We have checked whether the culture stage during which inhibitors are applied has an effect on plant regeneration. Moreover, for the first time in plants, we analyzed the effect of DNMT inhibitors on the ploidy of regenerants. The effect of AZA and ZEB on the global level of DNA methylation and the distribution patterns of methylated DNA in the protoplast-derived callus was also analyzed.

## Methods

### Plant material

As a plant material, two cultivars of *Brassica oleracea* var. *capitata fs. alba*, *‘*Kamienna Głowa’ (PlantiCo, Zielonki, PL) and ‘Kilaton F1’ (Syngenta, Basel, CH), were used. Seeds from both cultivars were surface disinfected as described by Kiełkowska and Adamus [42]. Disinfected seeds were air dried on filter paper and placed in 500 ml culture boxes with 80 ml of MS (Murashige and Skoog [43] medium (MS macro-, micro-elements and vitamins, 20 g/l sucrose, and 0.25% (*w/v*) Gelrite (Duchefa Biochemie, Haarlem, NL), pH 5.7–5.8, autoclaved (20 min, 121 °C, 0.1 MPa)). The cultures were kept under a 16-hour photoperiod supplied by fluorescent lamps (36 W Osram Biolux and Fluora) with a light intensity of 55 µmol m^− 2^ s^− 1^, at 25 ± 2 °C. The plants obtained as a result of the culture were further used as a protoplast source.

### Protoplast isolation and culture

Protoplasts were isolated from the leaves of 4- to 5-week-old seed-derived plants cultured in vitro. Leaves were cut into small pieces with the scalpel and subjected to plasmolysis, enzyme treatments and purification according to Kiełkowska and Adamus [42]. The density of cultured protoplasts was counted using a Fuchs-Rosenthal chamber (Heinz Herenz, Hamburg, DE) and adjusted to 8 × 10^5^ ml^− 1^ of culture medium.

Protoplasts were immobilized in filter-sterilized alginate according to the protocol of Kiełkowska and Adamus [44] using alginic acid sodium salt (Sigma-Aldrich, Poznan, PL) and calcium-agar medium. For alginate layers, 0.3 ml of the protoplast-alginate mixture was gently spread onto calcium-agar medium. The layers were transferred to the 60 × 15 mm Petri dish containing 4 ml of CPPO1 culture medium according Kiełkowska and Adamus [42] with modified PGR’s (0.45 µM 2,4-dichlorophenoxyacetic acid (2,4-D), 2.7 µM 1-naphtalenacetic acid (NAA), 2.2 µM 6-benzylaminopurine (BAP)), pH 5.6, filter-sterilized (pore size 20 μm, Millipore).

### Experimental setups

5-azacytidine (AZA, Sigma-Aldrich, item no. Z4775) and zebularine (ZEB, Sigma-Aldrich, item no. Z4775) were applied into protoplast culture in two experimental setups. In the first experiment, AZA or ZEB were added to the medium for protoplast culture at concentrations of 2.5, 5, and 10 µM. The treatment was applied for 10 days. An inhibitor-free medium was used as a control. After 10 days of treatment with each inhibitor and in control, the protoplast culture medium was replaced by inhibitor-free medium, and cultures were further incubated in the dark at 26 ± 2 °C for 3 weeks. The stocks of AZA and ZEB were prepared in double-distilled water. The medium supplemented with less stable AZA [45] was refreshed every 2 days, and the inhibitor was prepared freshly and kept on ice during preparation and before each use. This experiment was designed for the evaluation of the effects of applied DNMT inhibitors on protoplast-derived cell viability (1), cell wall regeneration (2), mitotic activity (3), micro-callus production (4), and the effectiveness of organogenesis (5).

The second experiment aimed at the evaluation of the direct effects of DNMT inhibitors on the regeneration process as well as the evaluation of the level and nuclear localization of DNA methylation. The protoplasts were cultured in inhibitor-free media, which was renewed once after 10 days. After 4 weeks of culture, the obtained protoplast-derived micro-calluses were transferred into solid regeneration medium and subjected to treatment with AZA and ZEB in concentrations of 25, 50, and 100 µM. The drug treatment lasted four weeks. To assure a fresh supply of AZA, calluses were transferred to the fresh medium with this drug every 5 days for the following 4 weeks of treatment. Here, the analyses of callus weight from the single alginate layer (1), effectiveness of organogenesis (2), global DNA methylation (3), and immunolocalization of 5mC (3) in the drug-treated and control cultures were performed.

### Microscopic observations of cultures

#### Viability and mitotic activity of protoplast-derived cells

Observations of cell viability and cell divisions were gathered from two Petri dishes from each treatment in each of three repetitions. Protoplast viability was evaluated by staining with a fluorescein diacetate (FDA) solution diluted in culture medium as described by Kiełkowska and Adamus [42]. Approximately 200 cells from single dish were evaluated. Viability was calculated as the number of protoplasts exhibiting green fluorescence per total number of observed cells (x100). Protoplast viability was evaluated on the first and fifth day after isolation. The frequency of division of protoplast-derived cells was calculated as the number of cells undergoing division and cell colonies per total number of observed cells/colonies (x100). Approximately 200 cells in different stages of division (including first and second mitosis and cell colonies) for each dish were evaluated. The frequency of divisions of protoplast-derived cells was observed on the fifth and fifteen days of culture. Observations were done under an Axiovert S100 inverted microscope (Carl Zeiss, Göttingen, DE) supplied with an HBO50 mercury lamp and filter set (λ_Ex_ = 485 nm, λ_Em_ = 515 nm).

#### Cellulose staining

The rate of cellulosic cell wall reconstruction in protoplasts was evaluated by staining with Fluorescent Brightener 28 (Calcofluor White M2R, Sigma-Aldrich, Poznan, PL). A 3.8 mM stock of calcofluor in water was used. Protoplasts were stained for approximately 10 min and observed under Axiovert S100 under filter set λ_Ex_ = 365 and λ_Em_ = 420. Photographs were made using the AxioVision Rel. 4.8. program (Zeiss) and a PowerShot G10 camera (Canon). The observations were done 48 h after culture establishment. Approximately 600 cells from each treatment was evaluated. The interpretation of the results was as follows: protoplasts that did not stain with Calcofluor White indicated that the cell walls had been completely degraded, with no marks of cellulose resynthesis; protoplasts with point-aligned whitish fluorescent signals indicated start points of cell wall rebuilding; and cells with the fluorescent signal evenly distributed over the cell surface indicated complete cell wall regeneration.

### Plant regeneration, acclimatization and ploidy analysis

After four weeks, cultures were observed under a stereomicroscope (Leica S6D, Leica Microsystems, Wetzlar, GE). Colonies approximately 0.5 mm in size and larger were counted on every alginate layer separately, and afterwards the cultures were subjected to regeneration. Calluses were released from Ca-alginate layers according to Kiełkowska and Adamus [16]. Regeneration was performed on solid P medium (MS [43] macro-, micro-elements, and vitamins with 5.4 µM NAA, 5.0 µM N^6^-(2-Isopentenyl)adenine (2iP), 0.06 µM gibberellic acid (GA_3_), 10 g/l sucrose, 20 g/l mannitol, and 0.25% (*w/v*) Gelrite (Duchefa), pH 5.7–5.8, autoclaved (20 min, 121 °C, 0.1 MPa)). Shoots developed on P medium were transferred for further development and rooting to MS hormone-free medium. The cultures, were transferred to fresh media every 4 weeks and were maintained at 25 ± 2 °C with a 16-hour photoperiod at a light intensity of 40 µmol m^− 2^ s^− 1^. The regeneration frequency was calculated as the number of shoots developed from callus per total number of callus cultured on the regeneration medium (x100). In first experiment, the effectiveness of organogenesis was evaluated after 16 weeks of culture on the regeneration medium. In the second experiment the analyses of callus weight (g) from the single alginate layer were done after 4 weeks of drug treatment. The effectiveness of organogenesis was evaluated after 16 weeks of culture on the regeneration medium.

Rooted plantlets from both experiments were planted into multipots filled with moistened coconut substrate (Ceres International Ltd., Pyzdry, PL) and transferred into the climatic chambers SANYO MLR-352 H (Sanyo Electric Biomedical Co. Ltd., JP) set up for 19 ± 2 °C with a 16-hour photoperiod, a light intensity of 45 µmol m^− 2^ s^− 1^, and an air humidity of 90%. The plants were acclimatized to *ex vitro* conditions for 2 weeks by a gradual reduction of the air humidity to the final value of 70%. Afterwards, the plantlets were replanted in 500-ml pots filled with garden soil (Hollas Group, Pasłęk, PL) and transferred to a room with a temperature of 20 ± 2 °C with a 16-hour photoperiod at a light intensity of 40 µmol m^− 2^ s^− 1^ for 4–6 weeks. The effectiveness of acclimatization was calculated as the number of plants adapted to *ex vitro* conditions per total number of plants subjected to acclimatization (x100).

The regenerants obtained from both experiments were subjected to ploidy analyses with flow cytometry. The ploidy was evaluated in plants just after acclimatization, before replanting and transferring plants to the greenhouse. The nuclei were released from the leaves, according to Kiełkowska et al. [46]. The analysis was done on Partec PA II (Partec, GmbH, DE).

### DNA isolation and quantification of global DNA methylation

The total genomic DNA was extracted from the 4-week-old callus treated with DNMT inhibitors in certain concentrations and controls (second experiment). The DNA extraction was done using the protocol of Adamus et al., [47]. DNA samples after isolation were rehydrated in 100 µL of deionized water. The DNA quantity was checked using a Nanodrop spectrophotometer (Nanodrop 2000, Thermo Scientific, Wilmington, US).

The Imprint^®^ Methylated DNA Quantification Kit (Sigma-Aldrich, item no. MDQ1) was used for the quantification of global DNA methylation according to the manufacturer’s instructions, using 150 ng of genomic DNA from tested samples and a methylated control DNA standard (positive control) supplied by the producer. The methylated DNA was detected using capture and detection antibodies and then quantified colorimetrically. The light absorbance was analyzed on a computer-controlled microplate reader (LEDETECT 96, Labexim Products, Lengau, AT) with dedicated software (Capture 96 Software, Biomed Dr. Wieser GmbH, Salzburg, AT). The experiment was performed with three biological (callus samples from independent culture experiments) and two analytical (DNA methylation colorimetric assays) replicates per treatment. The percent of methylation (quantities of 5-methyl-cytidine, 5mC) of the samples relative to the methylated control DNA were calculated according to the manufacturer’s formula using the single point method (Sigma-Aldrich, MDQ1 Product Information).

### 5mC immunostaining

The preparation of plant material (the 4-week-old callus treated with DNMT inhibitors and controls) and immunostaining were performed according to Braszewska-Zalewska and Hasterok [48]. Detection of cytosine methylation (5mC) was done using mouse antiserum raised against 5-methyl-cytosine (1:300 dilution in 1% bovine serum albumin (BSA) in phosphate buffered saline (PBS); Abcam cat. No. ab73938) and Alexa488 goat anti-mouse secondary antibody (Invitrogen, Molecular Probes cat. No. A-11001). Nuclei were counterstained with 4’,6-diamidyno-2-fenyloindol (DAPI). Images were captured separately for DAPI and Alexa 488 using dedicated filters. Images were registered using an Olympus FV1000 confocal system (Olympus, Tokyo, JP). Evaluation of 5mC signals distribution in callus nuclei was performed for a minimum of 100 nuclei per sample in three biological repetitions.

### Data collection and statistical analysis

In both tissue culture experiments, each treatment (accession × inhibitor type × inhibitor concentration, and controls) was represented by a minimum of ten Petri dishes. The experiment was repeated three times. Analyses of the collected data were performed using the ANOVA module. The mean separation was done according to Duncan’s multiple range test (MRT). All statistics were calculated with Statistica ver. 13.3 (TIBCO Software Inc.) software at a 0.05 probability level. The data were presented as a mean ± standard error of the mean (SEM).

## Results

### Results of experiment 1

In this experiment AZA and ZEB were applied directly to protoplast culture and the treatment was applied for 10 days.

#### Azacytidine induces dose-dependent reduction in protoplast-derived cell viability

**Fig. 1:**
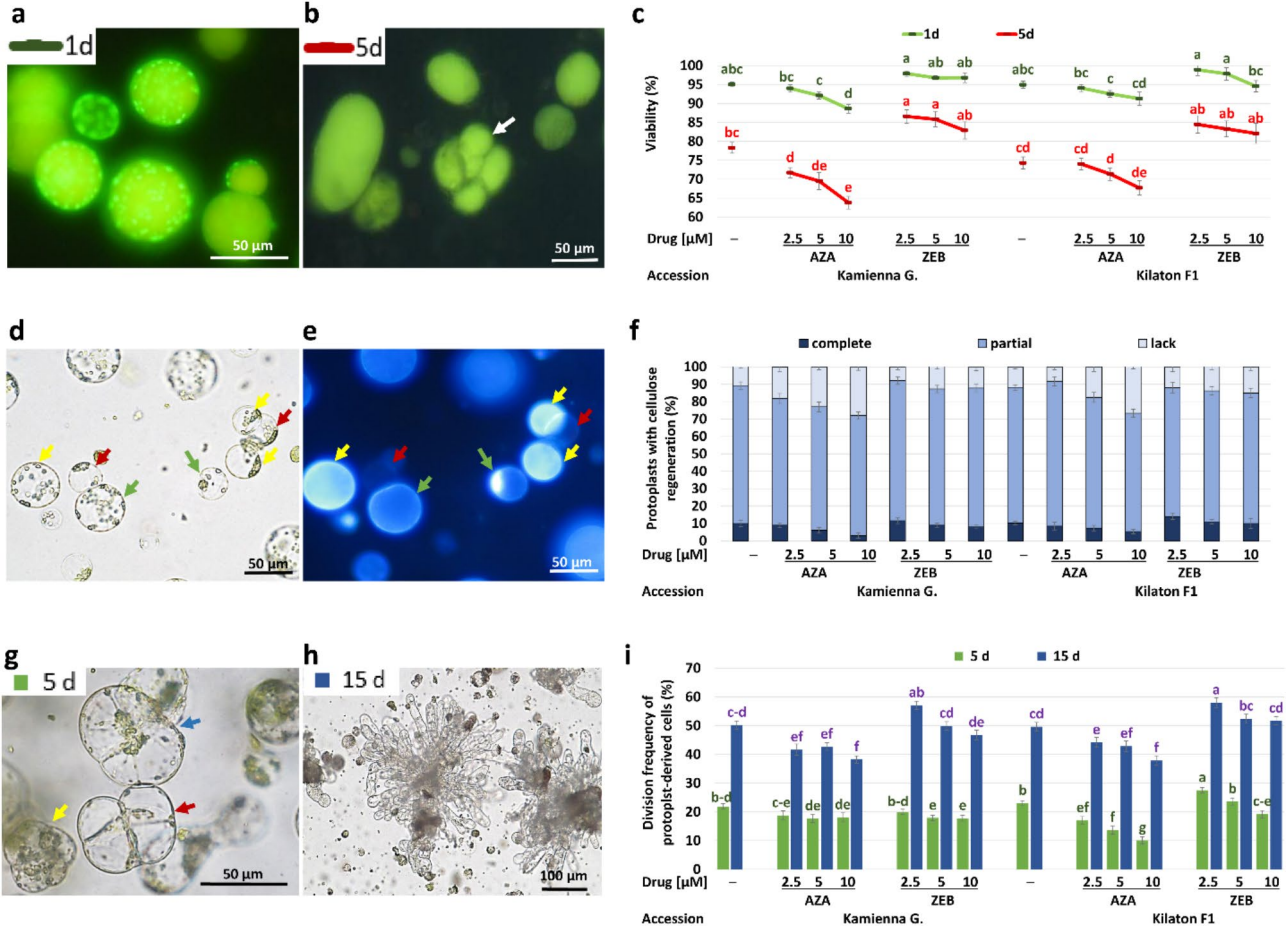
Development in protoplast cultures of cabbage treated with 5-azacytidine (AZA) and zebularine (ZEB) and in controls (─). Viability of protoplasts in 1st (**a**) and 5th day of culture (**b**). FDA-stained viable cells express yellow-green fluorescence after excitation with UV light. White arrow indicates cell colony. (**c**) Mean effect of accession, AZA and ZEB on the cell viability at 1st (green points) and 5th (red points) day of culture. Points are means ± SEM (*n* = 60). Points marked with the same letter are not significantly different (*P* ≤ 0.05, MRT). Statistical grouping for green and red points should be considered separately. Protoplasts stained with calcofluor and observed in white (**d**) and UV (**e**) light after 48 h of culture. Both pictures show the same field of view. Complete resynthesis (yellow arrows); partial resynthesis (green arrows); lack of cell wall synthesis (red arrows). Mean effect of accession, AZA and ZEB on the regeneration of cellulose in protoplasts (**f**). Bars are means ± SEM (*n* = 30). The division frequency of protoplast-derived cells in 5th day (**g**) and 15th day of culture (**h**). g: Enlarged cell with reorganized cytoplasm and chloroplasts before mitosis (blue arrow); cells after second mitosis (red arrow); few-celled colony (yellow arrow). h: Multiple-celled colonies; (**i**) Mean effect of accession, AZA and ZEB and its concentration on the division frequency of protoplast-derived cells in 5th and 15th day of culture. Bars are means ± SEM (*n* = 60). Bars denoted with the same letter are not significantly different (*P* ≤ 0.05, MRT). Statistical grouping for blue and green bars should be considered separately

On the first day of culture, the protoplasts were spherical with visible chloroplasts (Fig. 1a). Viability of protoplasts was not affected by cultivar (*p* = 0.80), but depended on the type and concentration of applied drugs and culture duration. The viability in controls was near 95% in both cultivars (Fig. 1c). In the cultures treated with AZA, a decrease in viability (from approximately 95% to 88–90%) was observed in parallel with an increasing concentration of the drug. In the cultures treated with 2.5 µM of ZEB, viability ranged from 97 to 98%, and after treatment with its highest concentration, it was similar (94–96%), independently of the cultivar.

On the fifth day of the culture, protoplast-derived cells changed their shape to more elliptic and elongated; moreover, a few-celled colonies were also detected (Fig. 1b, white arrow). At that time, the viability of cultured cells decreased in all tested treatments and in controls. The viability of cells treated with AZA decreased below the controls (74–78%) and was affected by the concentration of this drug. The viability of the cells treated with 2.5 µM of AZA was 71–74% and decreased to 64–67% after the application of 10 µM of this drug. At the same time, the viability of cells cultured on the media supplemented with ZEB was higher than that in the control cultures and was 84–86% in the cultures treated with 2.5 µM ZEB and approximately 82% in the cultures with 10 µM of this drug.

#### Azacytidine induces dose-dependent delay in cell wall reconstruction in protoplasts

The process of cellulose resynthesis was not synchronous. At 48 h of culture, we observed protoplasts that did not emit fluorescence (Fig. 1d-e, red arrows), indicating a lack of cellulose resynthesis. Some protoplasts showed point-aligned whitish fluorescent signals, indicating start points of cellulose resynthesis and cell wall rebuilding (Fig. 1d-e, green arrows). In other protoplasts, the fluorescent signal was evenly distributed over the protoplast surface, indicating complete cell wall regeneration (Fig. 1d-e, yellow arrows). The differences in the dynamics of cellulose regeneration in cabbage protoplasts were dependent on the presence of drugs in the medium, irrespective of the accession (Fig. 1f). In the control cultures of both cultivars, protoplasts with complete and partially reconstituted cell walls represented 10–11% and 78–79% of the observed cells, respectively. AZA-treated cultures showed a gradual decrease in the number of protoplasts with completely and partially regenerated cellulose, proportionally to the increased concentration of this drug in the medium. In the cultures treated with 2.5 µM of AZA, we observed in total 81–87% of cells with marks of cellulose resynthesis, while in the cultures treated with 10 µM of AZA, there were 71–74% of such cells. In the cultures treated with ZEB, we observed an overall higher number of protoplast-derived cells with regenerated cellulose compared to AZA treatment. In the cultures treated with 2.5 µM of ZEB, we observed in total 88–93% of cells with marks of cellulose resynthesis, which was slightly higher compared to the cultures treated with 10 µM of ZEB (85–88%).

#### Azacytidine induces dose-dependent reduction in mitotic activity of protoplasts, while zebularine in low concentration promotes mitoses

There was no difference in the mitotic activity of protoplast-derived cells among the tested cultivars (*p* = 0.27); however, it was affected by applied drugs. On the 5th day of culture, we observed structural changes in protoplast-derived cells. The majority of the cells changed their shape from spherical to oval, which was the morphological evidence of cell wall reconstruction. These cells were marked by reorganization of the cytoplasm and cell organelles, which preceded the first mitotic division (Fig. 1g, blue arrow). At the same time, we also observed cells undergoing first mitotic divisions and few-celled colonies (Fig. 1g, red and yellow arrows, respectively). In such early cultures, 22–23% of the cells in control medium underwent mitotic divisions (Fig. 1i). The mitotic activity AZA-treated cultures ranged from 10 to 19%. The protoplasts of ‘Kamienna Głowa’ were less sensitive to AZA treatments, and we observed similar mitotic activity (18–19%) irrespectively of the concentration of the drug. In ‘Kilaton F1’, the mitotic activity in AZA-treated cultures was lower (10–14%), than in control cultures (23%). Moreover, in this cultivar, we observed a decrease in the mitotic activity of protoplast-derived cells in parallel to the increasing concentration of AZA in the culture medium. The highest division frequency (27%), exceeding control (23%), was observed in ‘Kilaton F1’ on the medium containing 2.5 µM of ZEB. Higher concentrations of this drug decreased the mitotic activity of the protoplast-derived cells in this accession. In ‘Kamienna Głowa’, slightly higher (20%) mitotic activity was observed in the cultures treated with 2.5 µM of ZEB, compared to the division frequencies (18%) observed in the cultures treated with its higher concentrations.

The division frequency increased with the time of culture, and on average, 38–58% of protoplast-derived cells undergo divisions on the 15th day of culture (Fig. 1i). At that time, we observed undivided protoplasts, enlarged and plasmolyzed cells, as well as small, few-celled colonies and larger complexed star-like colonies composed of highly elongated cells (Fig. 1h). This last fraction was the most numerous among all structures undergoing cell divisions in the period when observations were taken. The division frequency in control cultures was approximately 50% in both cultivars. AZA treatment decreased division frequency (38–44%) below the control. In both cultivars, we observed similar mitotic activity (42–44%) among cells cultured on the medium supplemented with 2.5 and 5 µM of AZA, while 10 µM significantly decreased mitoses (38%). The highest frequency of cell divisions (57–58%) was observed in both cultivars in cultures treated with 2.5 µM of ZEB. Higher concentrations of ZEB caused a slight reduction in cell divisions (46–52%) in both cultivars.

#### Zebularine stimulates protoplast-derived micro-callus development

**Fig. 2:**
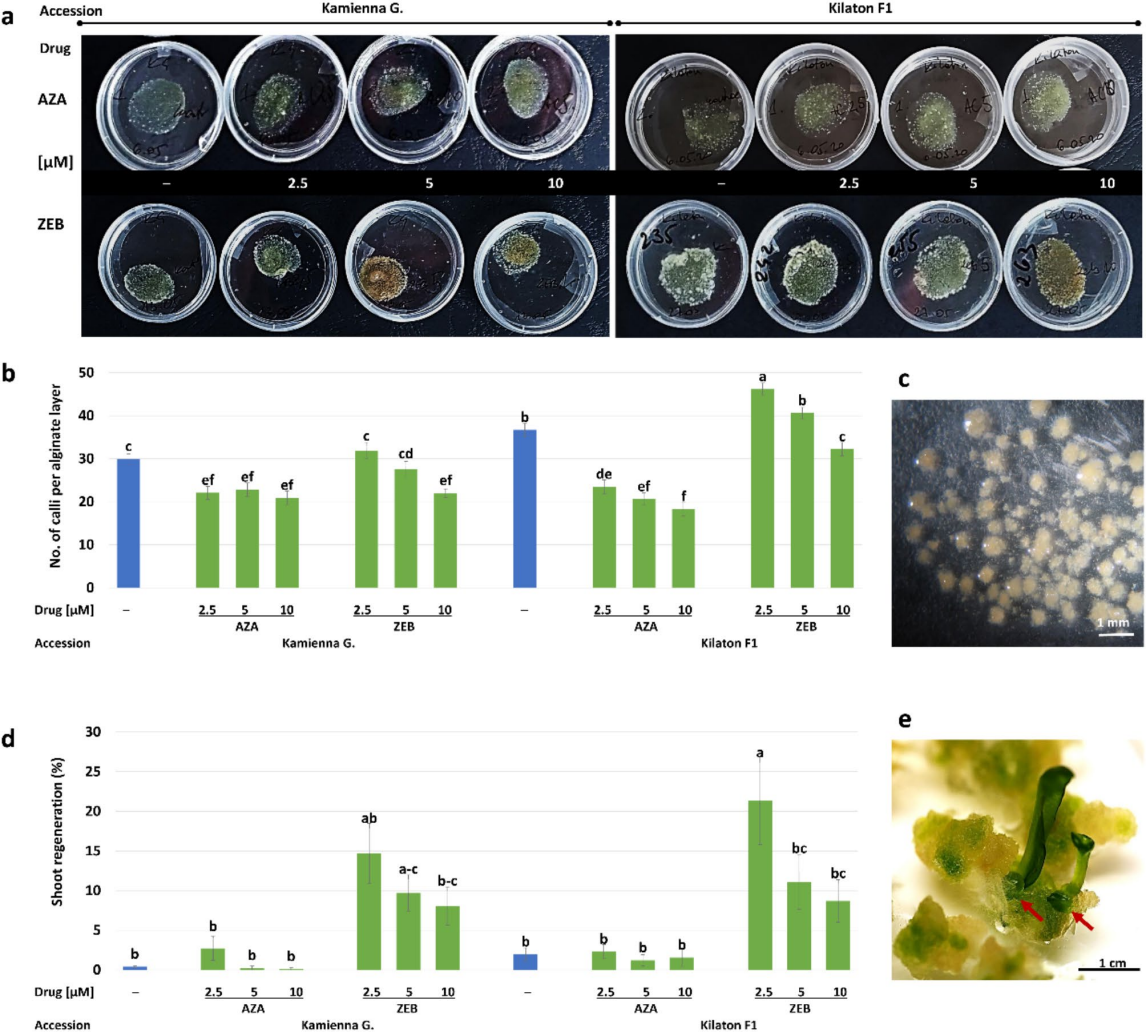
Micro-callus production and organogenesis from protoplast-derived cells of cabbage treated with 5-azacytidine (AZA) and zebularine (ZEB) and in controls (─). **(a**) Petri dishes with alginate layers overgrown with protoplast-derived callus after four weeks of culture. **(b)** Mean effect of accession, AZA, and ZEB and their concentrations on micro-callus production from protoplast cultures. Bars are means ± SEM (*n* = 45). Blue-colored bars represent controls. Bars denoted with the same letter are not significantly different (*P* ≤ 0.05, MRT). **(c)** Close up on alginate layer with micro-callus colonies. **(d)** Mean effect of accession, AZA, and ZEB and their concentrations on shoot regeneration. Bars are means ± SEM (*n* = 32). Blue-colored bars represent controls. Bars denoted with the same letter are not significantly different (*P* ≤ 0.05, MRT). **(e)** Shoot organogenesis (marked with arrows) from the protoplast-derived callus

Continuous mitotic divisions of protoplast-derived cells resulted in the formation of micro-calluses, visible macroscopically after four weeks of culture (Fig. 2a, c). Statistics showed an interaction between the accession and the drug on micro-callus development (Fig. 2b). The highest number of calluses (46 pcs per layer) was observed in cultures of ‘Kilaton F1’ supplemented with 2.5 µM of ZEB, and it exceeded the control (37 pcs per layer). The highest concentration of this drug caused a decrease in callus production. In ‘Kilaton F1’, the number of calluses decreased in parallel to the AZA concentration (average 18–23 per layer). In ‘Kamienna Głowa’, the highest number (32) of calluses per layer was observed in the cultures treated with the 2.5 µM of ZEB, and it was similar as in control cultures (30 calluses per layer). Higher concentrations of this drug caused a decrease in the number of calluses produced. In ‘Kamienna Głowa’, the treatment of the cultures with AZA lowered callus development (20–23 calluses per layer) compared to the control; however, there was no effect of AZA concentration on callus production.

#### Azacytidine and zebularine applied to protoplast culture medium enhance shoot regeneration and affect the ploidy of regenerants

Shoot development from protoplast-derived callus was observed in all experimental combinations (genotype × inhibitor type × inhibitor concentration); however, with different frequencies (Fig. 2d-e). The highest shoot regeneration (8–21%), increasing controls (0.5–1.4%) in both tested accessions, was observed from protoplast-derived calluses developed on the medium supplemented with ZEB. The highest number of shoots (21%) was regenerated from protoplast-derived callus colonies developed on the medium supplemented with 2.5 µM of ZEB. An increased concentration of ZEB in the culture medium had a negative effect on the regeneration process, and shoot regeneration was lower (8–11%). Regeneration from protoplast-derived calluses developed on the medium supplemented with AZA was similar to the control and did not increase by 3%. In total, from the first experimental setup, we obtained 762 shoots (Table 1).

**Table 1:** Acclimatization and ploidy analysis of regenerants derived from protoplast cultures cabbage treated with 5-azacytidine (AZA) and zebularine (ZEB). Ploidy: 2x – diploid, 4x – tetraploid, m/a – mixoploids and aneuploids (4x with chromosome losses)

Accession	Drug [µM]	Plants		Ploidy of regenerants			
		regenerated (no.)	acclimatized (no./%)	analyzed (no.)	2x (no./%)	4x (no./%)	m/a (no./%)
Kamienna Głowa	Control	4	4 (100)	4	4 (100)	0 (0)	0 (0)
	AZA 2.5	6	5 (83)	5	2 (40)	2 (40)	1 (20)
	AZA 5	7	7 (100)	7	2 (29)	4 (57)	1 (14)
	AZA 10	6	6 (100)	6	1 (17)	1 (17)	4 (67)
	ZEB 2.5	48	37 (77)	37	21 (57)	14 (38)	2 (5)
	ZEB 5	23	10 (43)	10	2 (20)	3 (30)	5 (50)
	ZEB 10	47	29 (62)	27	3 (11)	13 (48)	11 (41)
Kilaton F1	Control	66	53 (80)	53	48 (91)	5 (9)	0 (0)
	AZA 2.5	39	16 (41)	16	4 (25)	6 (38)	6 (38)
	AZA 5	17	12 (71)	12	4 (33)	6 (50)	2 (17)
	AZA 10	21	13 (62)	13	4 (31)	2 (15)	7 (54)
	ZEB 2.5	220	135 (61)	127	72 (57)	41 (32)	14 (11)
	ZEB 5	113	65 (58)	65	32 (49)	23 (35)	10 (15)
	ZEB 10	145	31 (21)	31	7 (23)	16 (52)	8 (26)
**Total**		**762**	**423 (55)**	**413**			

**Fig. 3:**
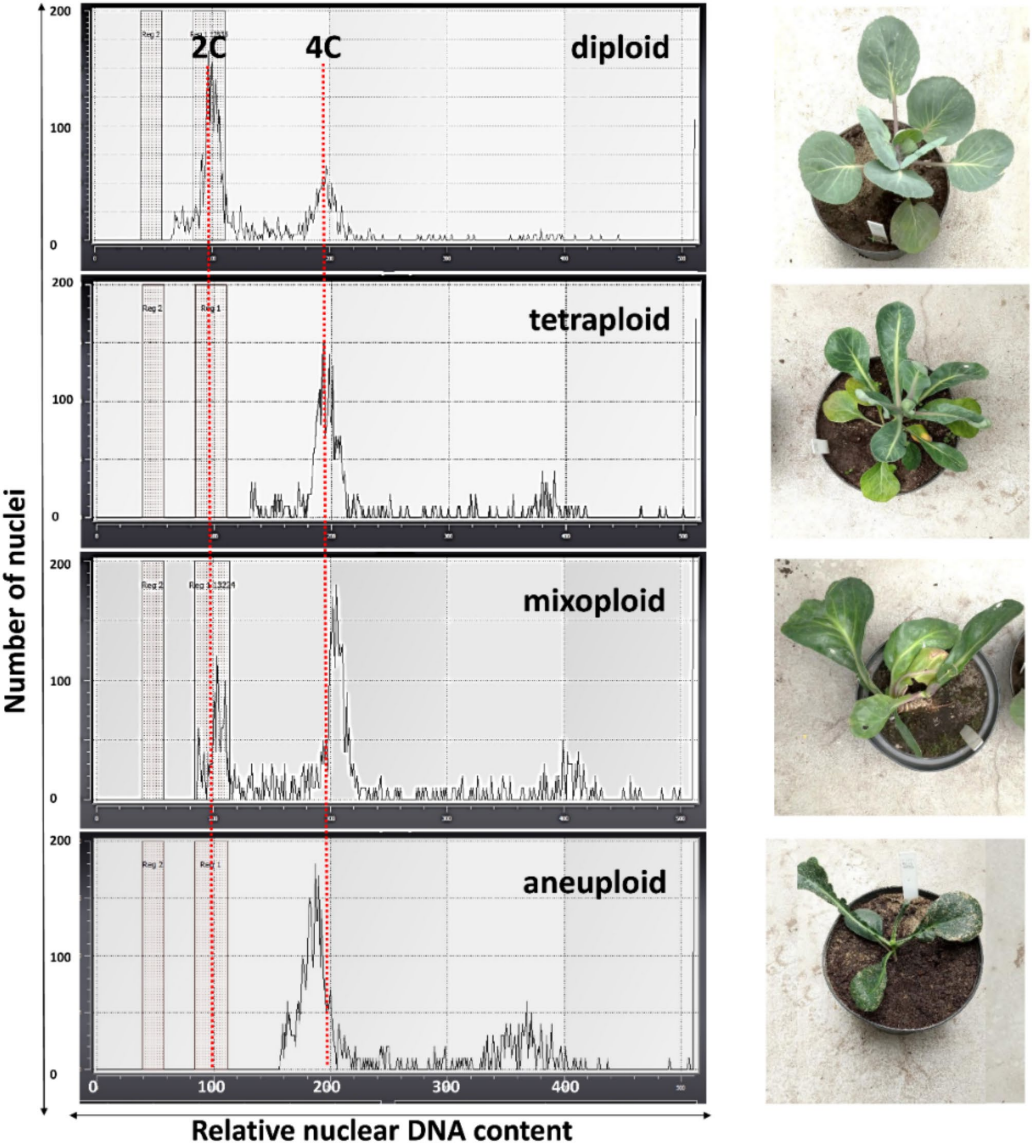
Flow cytometry histograms showing the relative nuclear DNA content of leaves of cabbage regenerants developed from control and drug-treated protoplast cultures, and exemplary pictures of regenerants of certain ploidy being approximately 3 months after acclimatization. On histograms, 2 C and 4 C peaks are marked by red dotted lines and refer to the DNA content of the control sample. Pictures from top: diploid (2x), tetraploid (4x), mixoploid (2x + 4x), and aneuploid (4x aneuploid) regenerants, respectively

The regenerated and rooted plants from the drug-treated and control combinations were subjected to acclimatization and then to ploidy analyses (Table 1; Fig. 3). The acclimatization rate was 80–100% for controls and 40–100% for regenerants obtained from inhibitor-treated cultures. The ploidy analysis revealed that regenerants from the control cultures were mostly diploids (91–100%). Amongst plants regenerated from drug-treated cultures, we observed a significant number of tetraploids, mixoploids (mainly 2x + 4x, but also 4x + 8x), and aneuploids. A higher number of tetraploids (17–57%) and mixoploids/aneuploids (14–67%) was observed among AZA-treated regenerants. The regenerants of both accessions obtained from the cultures treated with 2.5 µM of ZEB were mostly diploids (57%). The ploidy of the plants regenerated from the cultures treated with higher concentrations of ZEB varied, as a prevalence of tetraploids (30–52%) and mixoploids/aneuploids (15–50%) compared to diploids (11–49%) was observed.

### Results of experiment 2

In this experiment the protoplasts were cultured in inhibitor-free media until 4 week. The obtained protoplast-derived micro-calluses were transferred into regeneration medium and subjected to treatment with both AZA and ZEB for 4 weeks.

#### Proliferation of drug-treated protoplast-derived callus varies between tested genotypes

**Fig. 4:**
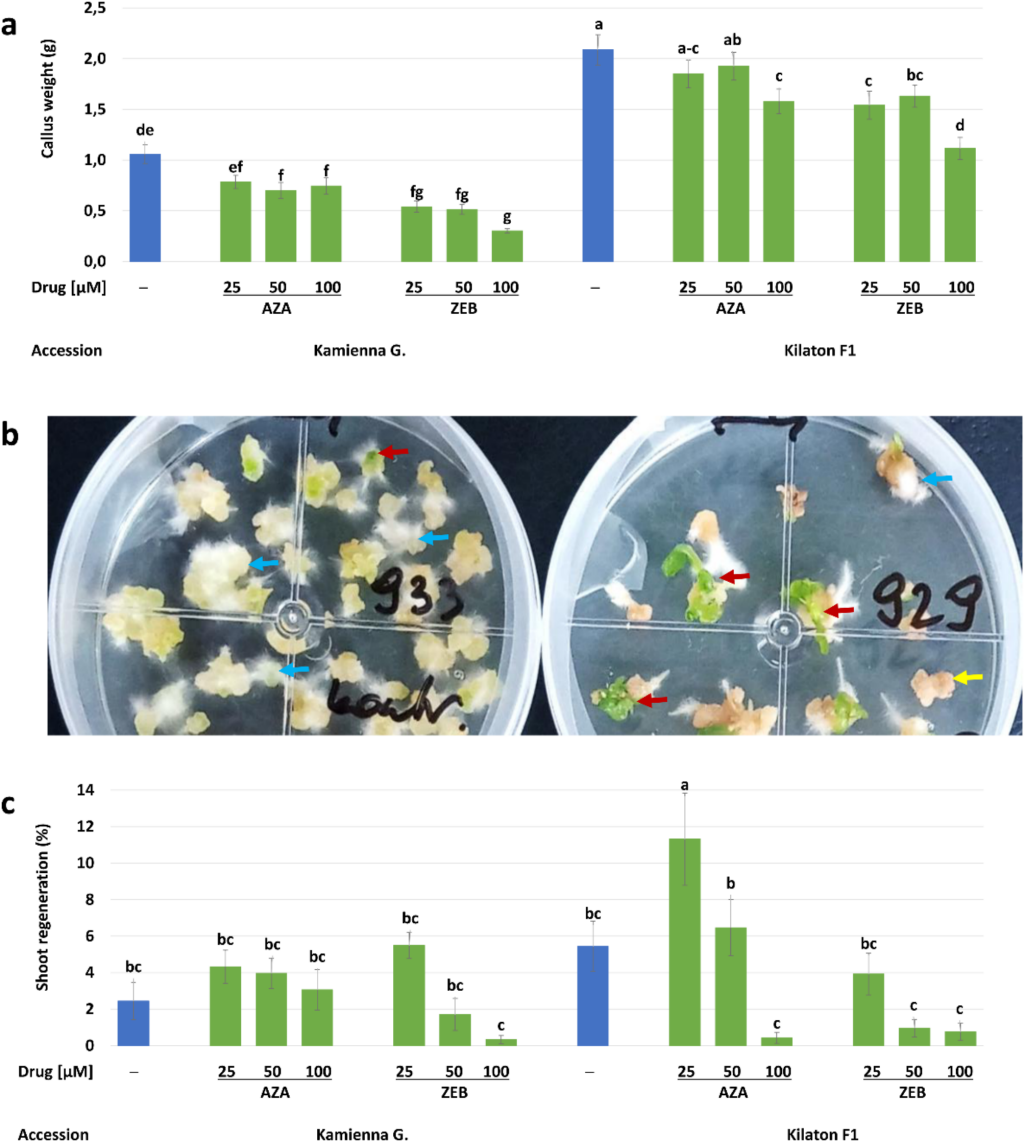
Development and shoot regeneration from protoplast-derived callus treated with 5-azacytidine (AZA) and zebularine (ZEB) and in controls (─). **(a)** The mean effect of accession and drugs on the weight of the callus. Bars are means ± SEM (*n* = 30). Blue-colored bars represent controls. Bars denoted with the same letter are not significantly different (*P* ≤ 0.05, MRT). **(b)** 90-mm petri dishes with calluses undergoing organogenesis in roots (blue arrows) and shoots (red arrows) and callus with no signs of organogenesis (yellow arrow). **(c)** Mean effect of accession and drugs on shoot regeneration after 16 weeks of culture. Bars are means ± SEM (*n* = 32). Blue-colored bars represent controls. Bars denoted with the same letter are not significantly different (*P* ≤ 0.05, MRT)

We observed an interaction of accession and the drug on the weight (g) of the drug-treated protoplast-derived callus (Fig. 4a). The mass of callus obtained in control cultures of Kilaton F1’ was higher (2.1 g/layer) compared to ‘Kamienna Głowa’ (1.1 g/layer). The treatments with both drugs caused a significant decrease in callus mass in ‘Kamienna Głowa’ compared to the control. The mean weight of the calluses treated with AZA was 0.7–0.8 g/layer, while in ZEB-treated samples it was slightly lower (0.3–0.5 g/layer). In ‘Kilaton F1’ the mass of callus treated with 25 and 50 µM of AZA was 1.8–1.9 g/layer and was similar to that in the control (2.1 g/layer). 100 µM of AZA caused a significant decrease in the weight of calluses (1.6 g/layer). The mean weight of calluses treated with ZEB was lower (1.1–1.6 g/ layer) compared to the control (2.1 g/layer).

#### Azacytidine applied to protoplast-derived callus enhance shoot regeneration and both drugs affect the ploidy of regenerants

After 16 weeks of regeneration of drug-treated calluses, we observed organogenesis in roots and shoots (Fig. 4b). Shoot development was observed in all experimental combinations (genotype × inhibitor type × inhibitor concentration); however, with different frequencies (Fig. 4c). Shoot regeneration from drug-treated calluses of ‘Kamienna Głowa’ was from 0.3 to 5% and was similar to the control (2%). In ‘Kilaton F1’, the highest shoot regeneration (11%) exceeding control (5%) was observed from calluses cultured on the medium supplemented with 25 µM of AZA. Shoot regeneration from calluses treated with 50 µM of AZA (6%) was similar to control (5%). The treatments with 100 µM of AZA caused a severe decrease in shoot organogenesis (0.4%). Shoot regeneration from calluses treated with 25 µM of ZEB was similar to the control and was 4%; however, higher concentrations of ZEB decreased regeneration (0.5%). In total, from the second experimental setup, we obtained 430 shoots (Table 2).

The average effectiveness of the acclimatization of rooted plantlets obtained from the second experiment was 53% (Table 2). The acclimatization rate was 66–100% for controls and 13–100% for regenerants obtained from drug-treated cultures. Ploidy analysis revealed that regenerants from the control cultures were mostly diploids (91–96%); however, single (1–3%) plants identified as tetraploids were observed. The highest number (60%) of diploids was observed among plants regenerated from calluses of ‘Kilaton F1’ treated with 25 µM of ZEB and in ‘Kamienna Głowa’ treated with 50 µM of ZEB (54%). In general, the majority of regenerants (up to 71%) obtained from the calluses treated with both drugs were tetraploids, mixoploids, and aneuploids. In both accessions, among plants regenerated from the calluses treated with 100 µM of both drugs, only tetraploids and mixoploids were observed. A higher number of plants (17–71%) with altered ploidy was observed among AZA-treated regenerants.

**Table 2:** Acclimatization and ploidy analysis of plants obtained from the protoplast-derived callus of cabbage treated with 5-azacytidine (AZA), zebularine (ZEB) and in controls. Ploidy: 2x – diploid, 4x – tetraploid, m/a – mixoploids (mainly 2x + 4x) and aneuploids (4x and 8x with chromosome losses)

Accession	Drug [µM]	Plants		Ploidy of regenerants			
		regenerated (no.)	acclimatized (no./%)	analyzed (no.)	2x (no./%)	4x (no./%)	m/a (no./%)
Kamienna Głowa	Control	26	26 (100)	26	25 (96)	1 (4)	0 (0)
	AZA 25	36	29 (80)	29	5 (17)	19 (66)	5 (17)
	AZA 50	25	14 (56)	14	4 (29)	4 (29)	6 (43)
	AZA 100	30	4 (13)	3	0 (0)	1 (33)	2 (67)
	ZEB 25	39	11 (28)	11	1 (9)	8 (73)	2 (18)
	ZEB 50	14	11 (79)	11	6 (54)	1 (9)	4 (36)
	ZEB 100	2	2 (100)	2	0 (0)	1 (50)	1 (50)
Kilaton F1	Control	76	50 (66)	45	42 (93)	3 (7)	0 (0)
	AZA 25	85	57 (67)	45	20 (44)	20 (44)	5 (11)
	AZA 50	40	7 (17)	7	1 (14)	5 (71)	1 (14)
	AZA 100	2	2 (100)	2	0 (0)	1 (50)	1 (50)
	ZEB 25	39	5 (13)	5	3 (60)	1 (20)	1 (20)
	ZEB 50	7	3 (43)	3	0 (0)	1 (33)	2 (67)
	ZEB 100	9	6 (67)	6	0 (0)	3 (50)	3 (50)
**Total**		**430**	**227 (53)**	**210**			

#### AZA and ZEB induced hypomethylation and modulated distribution patterns of methylated DNA in protoplast-derived callus

**Fig. 5:**
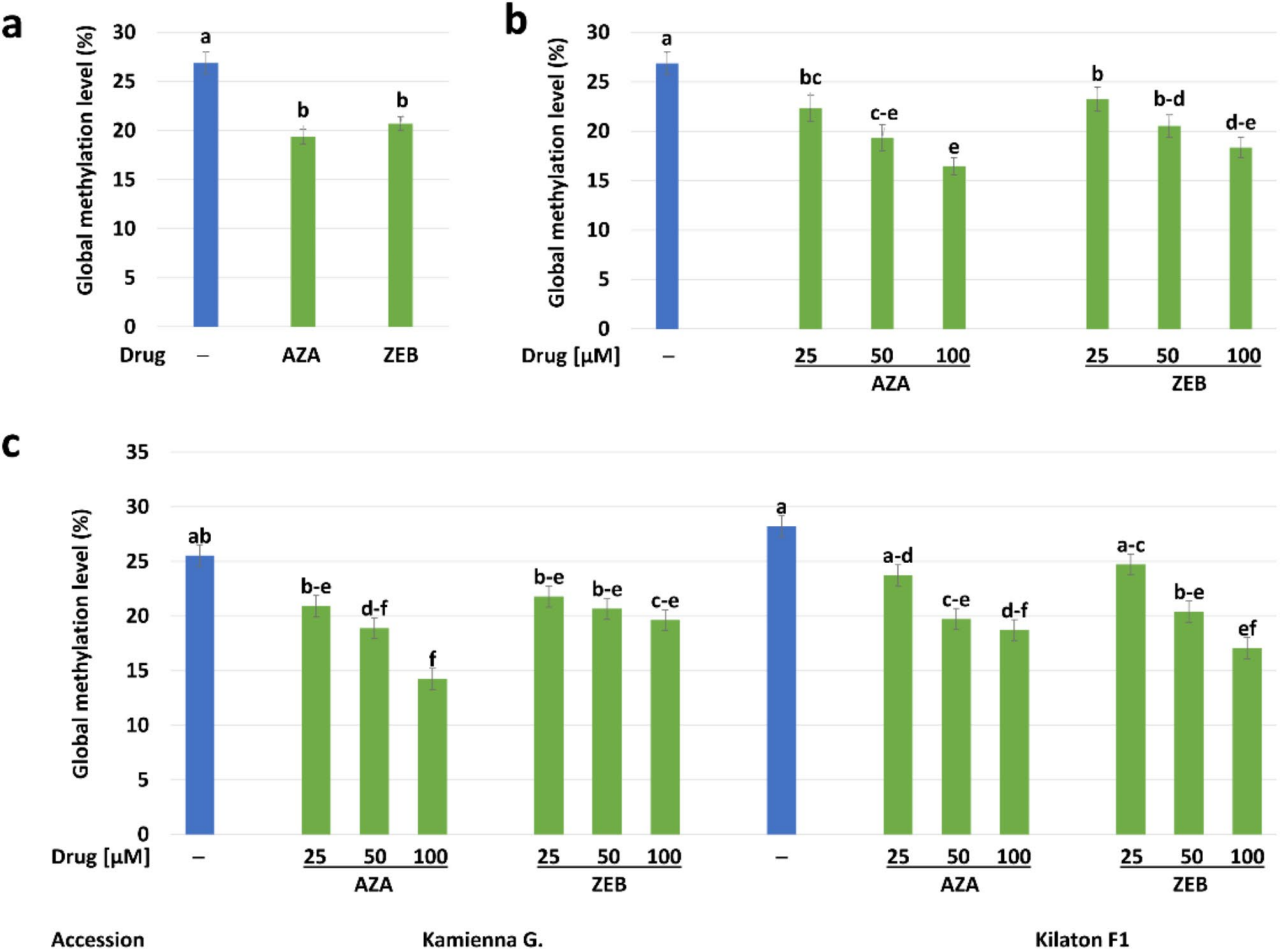
Quantification of global levels of DNA methylation and immunolocalization of 5-methyl-cytidine (5mC) in nuclei of 4-week-old protoplast-derived calluses of cabbage treated with 5-azacytidine (AZA) and zebularine (ZEB) and in controls (─). Mean effect of: the inhibitor **(a)**, the inhibitor × concentrations **(b)**; the inhibitor × concentrations × accession **(c)** on global levels of DNA methylation. Bars are means ± SEM (*n* = 8). Blue-colored bars represent controls. Bars denoted with the same letters are not significantly different (*P* ≤ 0.05, MRT)

We observed a decrease in the global level of DNA methylation in samples treated with both drugs (19–21%) compared to untreated controls (27%). Moreover, both drugs decreased the overall DNA methylation in tested callus samples to similar levels (Fig. 5a). The decrease in DNA methylation level in drug-treated samples was concentration-dependent; however, a higher decrease was recorded in AZA-treated samples (Fig. 5b). A detailed analysis of factors (Fig. 5c) showed differences in DNA methylation levels in control calluses of both cultivars (‘Kamienna Głowa’ 26% and ‘Kilaton F1’ 28%). AZA-treated calluses of ‘Kamienna Głowa’ had lower levels of DNA methylation in comparison to the control. The DNA methylation level in samples treated with 25 µM of AZA was 21%, while its higher concentrations decreased it (14–19%). Samples of ‘Kamienna Głowa’ treated with ZEB had similar DNA methylation levels (19–22%) irrespective of drug concentration. In ‘Kilaton F1’, the samples treated with 25 µM of AZA had a DNA methylation level slightly decreased (24%) compared to the control (28%). Samples of this cultivar treated with higher concentrations of AZA had DNA methylation levels reaching 19–20%. In samples treated with 25 µM of ZEB, the levels of DNA methylation were slightly lower (25%) than in the control, and higher concentrations of this drug caused a gradual decrease in methylation levels.

**Fig. 6:**
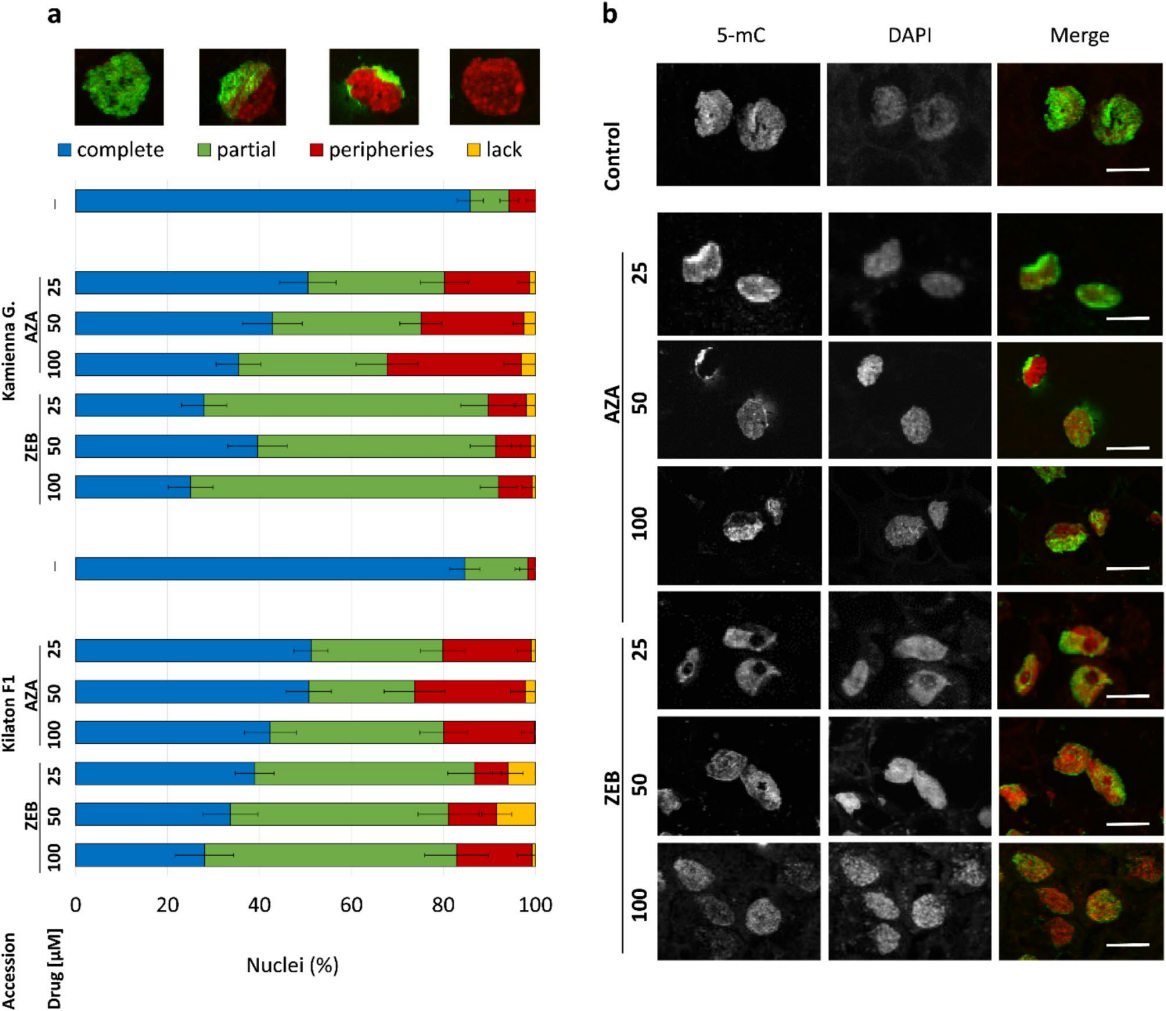
Immunolocalization of 5-methyl-cytidine (5mC) in nuclei of callus samples treated with 5-azacytidine (AZA) and zebularine (ZEB) and in controls (─). (**a**) Qualitative analysis of 5mC immunosignals. Bars are means ± SEM (*n* = 12). Miniatures show a representative distribution of 5mC patterns (green signals) in nuclei in each observed category. (**b**) In situ localization of 5mC immunosignals (green in merge). Nuclei were counterstained with DAPI (red in merge). All bars in the merge = 5 μm

Immunofluorescence assays showed that the 5mC immunosignals covered nuclei evenly and profusely in 85% of the control samples of both cultivars (Fig. 6a-b). In the nuclei of callus samples treated with both drugs, the pattern was different than in controls. We detected the 5mC immunosignals in specific areas of the nuclear surface (partial distribution) or exclusively on the nuclear peripheries. In 23–37% of nuclei in cells of AZA-treated calluses, 5mC immunosignals were partially distributed, and in 18–29% of nuclei, signals were located at peripheries. Across both cultivars, the number of nuclei with a partial and peripheric distribution of 5mC immunosignals increased in parallel to the drug concentration. In comparison to control samples, 5mC immunosignals in the nuclei of samples treated with ZEB were more scattered and covered mostly partial regions. Partial distribution of 5mC immunosignals was observed in nuclei of 51–67% of tested samples in this combination.

## Discussion

Initial experiments in plants showed that drug-induced DNA hypomethylation promotes the reprogramming of gene expression, the acquisition of totipotency, and the initiation of embryogenesis [17, 19, 29, 38]. These results were a prerequisite for our study on protoplasts. To the best of our knowledge, this is the first report comparing the effects of AZA and ZEB applied to plant protoplast cultures at different stages of their development and highlighting their effect on whole genome stability.

Our results showed that both used DNMT inhibitors display dose-dependent toxicity to protoplast cultures reflected in decreased cell viability; however, ZEB showed much less toxic effect compared to AZA (Fig. 1c). Studies on animal cells showed that the toxicity of DNMT inhibitors is mediated by DNA double-strand break covalent trapping of DNA methyltransferase and apoptosis rather than by the DNA demethylation process itself [49–52]. DNA breaks caused by DNMT inhibitors were also reported in plants [38, 53] but the nature and the extent of the DNA damages are currently unknown. Moreover, we observed that both inhibitors have a concentration-dependent effect on cell division, and ZEB was more effective in stimulating and maintaining mitoses in protoplast-derived cells compared to AZA (Fig. 1i). Studies on microspore cultures showed that AZA in dosages of 1.25–2.5 µM can stimulate cell proliferation, while higher concentrations lead to cell death [17, 19]. In lettuce, division efficiencies of protoplast-derived cells were similar, regardless of AZA concentrations (0.01–1 µM) [21]. In our study, 2.5 µM of AZA decreased mitoses of protoplast-derived cabbage cells below controls, but the same concentration of ZEB promoted mitoses above controls. Moreover, protoplast-derived calluses proliferated after treatments with 25–100 µM of AZA and ZEB while in other study in larch, 100 µM of AZA decreased the callus growth [54]. Summarizing above, protoplasts exhibit greater susceptibility to DNMT inhibitor treatments than microspores and calluses, making the selection of the optimal concentration of drugs for a certain species and a specific explant essential.

Here, we tracked the dynamics of cell wall rebuilt in drug-treated and control protoplasts (Fig. 1f). Cell wall reconstruction occur at the first stages of protoplast development, allowing further mitotic divisions and differentiation [16]. AZA-treated cultures showed a gradual decrease in the number of cells with completely and partially resynthesized cellulose, proportionally to the increased concentration of this drug in the medium. In the cultures treated with ZEB, we observed an overall higher number of protoplast-derived cells with completely and partially resynthesized cellulose compared to AZA treatment. Thus, it seems that AZA induces dose-dependent delay in cell wall reconstruction in protoplasts. There are no other studies showing the effect of neither AZA nor ZEB on cell wall regeneration in protoplasts; however, the link between cell wall assembly and DNA demethylation drugs was observed in *Beta vulgaris* cell lines [55] and *Brachypodium distachyon* callus [56]. In sugar beet, lignification and the incorporation of cell wall-bound phenolic compounds were altered by treatments with 10–1000 µM of AZA or DAC. In *Brachypodium*, treatments with 5 or 50 µM of AZA resulted in lack of extensin epitope immunodetection in callus samples. Extensins are glycoproteins essential for cell wall assembly and growth by cell extension and expansion in higher plants [57]. The proper cell wall assembly is essential to actively growing cells; therefore, any disruptions to this process are detrimental, as they can result in cell death ultimately [58].

We further analyzed the effects of AZA and ZEB on micro-callus formation from protoplast cultures, which is an important step preceding plant regeneration. More protoplast-derived micro-calluses were produced in cultures treated with ZEB compared to AZA; however, the effect was concentration-dependent (Fig. 2b). There are no other studies showing the effect of ZEB on protoplast-derived micro-callus production; however, treatment (1 µM) with the histone deacetylase inhibitor Trichostatin A (TSA) increased micro-callus formation and callus development in *Arabidopsis* protoplasts [59]. Choi et al. [21] showed that AZA (0.1 and 1 µM) promoted callus proliferation from lettuce protoplasts. Above results imply that the chemicals causing epigenetic modifications might stimulate protoplast-derived cell division, and if the mitoses are undisturbed, they lead to increased micro-callus formation.

The low frequency of plant regeneration is one of the main limitations of the routine application of protoplast technologies in crop improvement [42, 60–62]. The effects of chemical DNA demethylation on the regeneration from protoplast culture have not been studied so far. The only available study demonstrated that the addition of TSA and AZA to protoplast culture medium promoted protoplast-derived callus formation; however, plant regeneration was not reported [21]. Our study clearly showed that the period in which treatments with DNMT inhibitors are applied to protoplast cultures plays a crucial role in the regeneration process. We observed higher efficiency of regeneration when drugs were applied to protoplasts (Experiment 1, Fig. 2d)) compared to the treatment applied to protoplast-derived micro-calluses (Experiment 2, Fig. 4c). This aligns with observations in soybeans where the treatment of cotyledon explants with 10–50 µM of AZA during the second week of cultivation enhanced shoot regeneration; however, its earlier (1st week) or extended application (in 1st and 2nd week) reduced regeneration [63]. In contrast, there are very few studies showing the effect of ZEB on plant cell development and regeneration in vitro. In *Arabidopsis*, application of 40 and 80 µm of ZEB caused reversible growth retardation in seedlings [40]. In cotton, 100 µm of ZEB resulted in somatic embryo formation from non-embryogenic callus [64]. Here, we reported the first study on the effect of ZEB on organogenesis in vitro in plants. Our results showed that ZEB is much more effective in stimulating organogenesis from protoplast-derived callus compared to AZA, especially when it is supplied directly to protoplasts. More studies are needed in this regard; however, the supplementation of the culture media with epigenetic regulators could be potentially used as a new alternative to increase the regeneration efficiency of higher plants. Importantly, as the changes in chromatin structure during callus proliferation and *de novo* organogenesis are different [65, 66], the effects of epigenetic regulators on each step of plant regeneration will differ and must be verified experimentally for each species and explant type.

We have also evaluated the ploidy level of the regenerants obtained from DNMT inhibitor-treated and control cultures. We obtained similar results from both conducted experiments and both tested accessions. An increased number of plants with altered ploidy (tetraploids, mixoploids, and aneuploids) was observed among regenerants obtained from AZA-treated cultures, compared to ZEB-treated cultures. Moreover, we observed alterations in the appearance (malformed leaves, dwarfed habitat, and darker leaf coloration) of the regenerated plantlets derived from cultures treated with both DNMT inhibitors compared to control plants. Unfortunately, available papers on the effect of DNMT inhibitors on microspore cultures [17, 19], somatic embryogenesis [18, 28, 64] and callus proliferation [21, 54, 56] did not report the ploidy status of the obtained regenerants. The possible reason behind the observed changes in ploidy and morphology in our study might be a drug-induced genome instability. It has been shown in yeasts and in mammal cells that AZA increased the rate of chromosome aberrations (chromatid breaks, chromosome fragmentation) [67, 68], and micronuclei formation [67] but also endoreduplications and megakaryocytic cells [67, 69–71]. In plants, these studies are sparse, but it has been reported that 40–100 µM of AZA caused chromosome breakage in wheat root tips [72], growth retardation and changes in the flowering time and flower sex in flax [73] and wild potato [74]. Cauliflower, broccoli, and ‘rapid-cycling’ brassica seedlings treated with AZA (300–1500 µM) exhibited dwarfism, bushiness, and sterility [75]. It has also been shown, that 100–250 µM of ZEB induced chromosomal aberrations in wheat [76], triticale [77], and *Arabidopsis* [78]. Recent studies demonstrated cell cycle arrest in ZEB-treated breast cancer cells [79], what might result in polyploidy [80]; however, further research in plants in this regard is needed.

We observed a significant decrease in global methylation level in samples treated with DNMT inhibitors compared to controls. Our estimates of relative DNA methylation loss show that higher concentrations of AZA may have a larger effect than ZEB, whereas at lower concentrations, the difference in action of both inhibitors is less clear. This aligns with findings in *Arabidopsis* seedlings, where AZA and ZEB caused a similar decrease in genome-wide DNA methylation [53]. Considering obtained results and the fact that AZA is less stable than ZEB [29], it seems that AZA may have a greater initial effect that persists over subsequent mitoses, and ZEB incorporates less frequently into the DNA double helix or binds less strongly to DNA methyltransferases [34, 53, 81]. Moreover, it has been reported that the primary DNA repair pathways activated in ZEB- and AZA-treated plant samples differ. Nucleotide excision is the dominant pathway in the repair of AZA-induced DNA damage, while homologous recombination (HR) was found to mediate ZEB-induced damage repair [81]. Any difference in the rate at which these nucleotide analogs are removed from the DNA helix may contribute to a difference in the amount of inhibitor-caused demethylation. Our study also revealed differences in the pattern of 5mC immune signals of drug-treated samples compared to untreated controls, what was also observed in AZA-treated microspores *B. napus* [17] or triticale [19]. Such results illustrate that both AZA and ZEB treatments affect the global distribution pattern of methylated DNA in the plant cell nucleus, which further affects subsequent stages of cell development, including whole plant regeneration and the genome stability of obtained regenerants.

## Conclusions

Epigenetic and cancer research widely uses DNMT inhibitors, but we still don’t fully understand their biological effects and the mechanism(s) of action in plants. Our results showed that AZA applied to protoplast culture medium induced a dose-dependent reduction in protoplast viability, delayed cell wall reconstruction in protoplasts, and induced a dose-dependent reduction in mitotic activity of protoplasts, while ZEB promoted mitoses-stimulated protoplast-derived micro-callus development. We observed that the period in which the treatments with inhibitors of DNA methylation are applied plays a crucial role in the regeneration process. DNMT inhibitors added to protoplast culture medium more effectively promoted shoot organogenesis compared to cultures where inhibitors were added directly into the solid regeneration medium. Furthermore, for the first time in plants, we demonstrated that the treatments with DNMT inhibitors induce polyploidization. This is particularly interesting as DNMT inhibitors were suggested as stimulators of microspore embryogenesis [17, 19], and somatic embryogenesis [18, 28, 64]. While DNMT inhibitors-induced polyploidization might be considered beneficial during microspore embryogenesis, as it might result in genome doubling of a haploid cell, this process during somatic embryogenesis aiming at conservation of the original genome in the course of micropropagation might be problematic. Our study also revealed, that both inhibitors caused overall similar effects on DNA methylation; however, we observed differences in the magnitude of DNA methylation loss that existed between AZA- or ZEB-treated callus samples. Comparison of the data on global methylation levels with the regeneration efficiency suggests that organogenesis in *B. oleracea* is partially controlled by variation of DNA methylation levels. Moreover, strong reduction in the level of DNA methylation, caused by high concentrations of applied inhibitors, adversely affected the organogenesis process.

## Data Availability

The datasets supporting the conclusions of this article are included within the article or are available from the corresponding author on reasonable request.
